# Hemorrhagic Cholecystitis after Warfarin Use for Deep Vein Thrombosis

**DOI:** 10.1055/s-0038-1660450

**Published:** 2018-06-18

**Authors:** Eric Donn, Ian Atkinson, Andrew McCague

**Affiliations:** 1 Natividad Medical Center, Salinas, California

**Keywords:** hemorrhagic cholecystitis, warfarin, anticoagulation, cholecystitis, deep vein thrombosis

## Abstract

Hemorrhagic cholecystitis is an uncommon form of acute cholecystitis which can be rapidly fatal. It may be hard to detect as it frequently presents with symptoms found in other, more common diagnoses. We report the case of a 63 year old man recently started on anticoagulation for deep vein thrombosis who was found to have hemorrhagic cholecystitis.

Hemorrhagic cholecystitis is an uncommon form of acute cholecystitis which can be rapidly fatal. It may be hard to detect as it frequently presents with symptoms found in other, more common diagnoses. Here, we report the case of a 63-year-old man recently started on anticoagulation who initially presented with suspicion for pneumonia versus pulmonary embolism, but was found to have hemorrhagic cholecystitis instead.

## Case Report

**Fig. 1:**
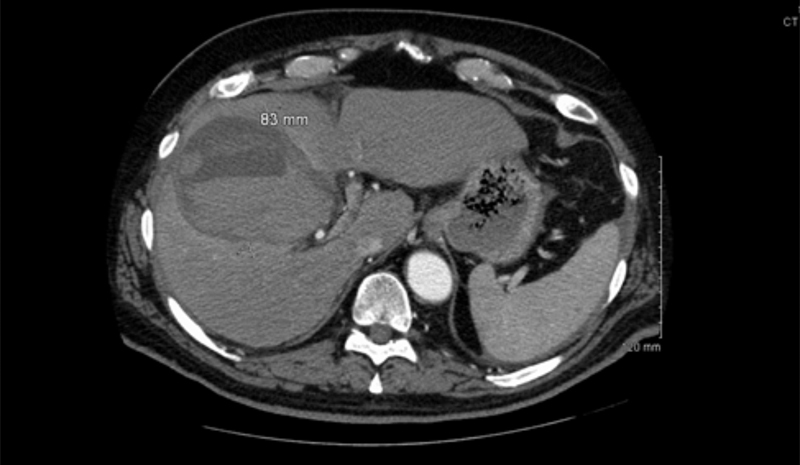
Computed tomography (CT) of abdomen and pelvis showing distended hemorrhagic appearance of gallbladder, no stones.

A 63-year-old man with a deep vein thrombosis diagnosed 5 days prior, presented with fever, tachycardia, and nausea/vomiting. The patient had a past medical history of cerebrovascular accident 9 years prior with residual left-sided weakness, epilepsy, hypertension, and hyperlipidemia. The patient denied smoking, alcohol, or drug use. The patient was transferred from an acute rehab center where a left lower extremity deep vein thrombosis had been found on ultrasound 5 days prior, and anticoagulation started. He was bridged from enoxaparin to oral warfarin. Initial laboratories were as follows: white blood cell count 12,600/mL ^3^ , hemoglobin 14.0 g/dL, platelet 302,000/mL ^3^ , total bilirubin 2.1 mg/dL, aspartate aminotransferase 68 IU/L, alanine aminotransferase 56 IU/L, prothrombin time 20.3 seconds, international normalized ratio (INR) 1.95, albumin 3.1, fibrin degradation products > 10 and < 40, blood urea nitrogen 14, and serum creatinine 0.83. Initial physical exam showed left lower lobe decreased breath sounds, and an unremarkable abdominal exam. At that time, the patient denied any pain, current nausea/vomiting, shortness of breath, constipation, or diarrhea. Chest X-ray suggested consolidation in the left lower lobe. The decision was made to admit the patient as he met the criteria for systemic inflammatory response syndrome, possibly due to a left lower lobe pneumonia versus pulmonary embolism. For further evaluation, a chest computed tomography (CT) scan was ordered. While the CT scan showed no evidence of pulmonary embolism or pneumonia, it did incidentally reveal a distended appearance to the gallbladder ( Fig. 1 ). Ultrasound showed distended gallbladder with a heterogeneous mixture of intermediate and low echogenic material with no evidence of stones, which was concerning for possible pus or hemorrhage. Liver was of normal size and echogenicity. Patient was started on ceftazidime, clindamycin, and vancomycin empirically for systemic inflammatory response syndrome/sepsis. The patient was initially ruled out as a surgical candidate due to his elevated INR, and given his extensive deep vein thrombosis, it was felt that the risk of stopping anticoagulation outweighed the benefits and he was recommended for interventional radiology (IR)-guided cholecystostomy. Repeat physical exam on hospital day #2 revealed a firm, tender right upper quadrant of the abdomen, with all other quadrants being soft and nontender. The patient's INR spiked to 6.28 on hospital day #2 and his hemoglobin dropped to 9.8 g/dL. This elevation of his INR was attributed to his continued anticoagulation, which was subsequently discontinued. INR on hospital day #3 was 4.53, and 4.94 on hospital day #4, at which point vitamin K and fresh frozen plasma were administered. INR improved to 1.69 on hospital day #5. IR-guided cholecystostomy was attempted on hospital day #4. The decision to proceed with an IR-guided approach had been made earlier as it was felt he was at high risk for surgery and septic. However, there was an organized clot with no drainable material and subsequently no drain left in place. An inferior vena cava filter was placed on hospital day #8. The family and patient had also up to this point refused surgery due to his history of stroke, do not resuscitate/do not intubate status, and their perceived risk of surgery. However, they eventually agreed to have surgery. On hospital day #11, laparoscopic cholecystectomy was performed, and revealed dense adhesions surrounding the gallbladder including the omentum and bowel. These adhesions were taken down to reveal a very large distended gallbladder with areas of necrosis and perforation. The contents of the gallbladder contained approximately 1,000 cubic centimeters of old clot which had perforated near the infundibulum with clot extending out into Morison's pouch and the right upper quadrant causing dense adhesions to the liver. The gallbladder was freed from these adhesions and the clot was removed. The gallbladder was dissected and freed from surrounding adhesions. The cystic duct was secured with a “PDS Endoloop” made by Ethicon. During the course of the operation, patient experienced significant bleeding and required four units of packed red blood cells. The operation was converted to an open cholecystectomy to obtain hemostasis, and a Jackson-Pratt drain was left in place. Pathology of the gallbladder specimen showed extensive hemorrhage, acute inflammation, and necrosis. Pathology of a liver specimen showed moderate fibrosis, which was suggestive of cirrhosis. However, hepatitis serology was all negative, and ultrasound of the liver showed a liver of normal size and echogenicity. No other investigation showed signs of chronic liver disease. Patient had one episode of bleeding from his drain which required transfusion, but the remainder of his hospital course was otherwise uneventful. The patient was considered too high risk for further anticoagulation and was discharged with an inferior vena cava filter in place.

## Discussion

Hemorrhagic cholecystitis is a rare disease but has been previously associated with blunt trauma, anticoagulation, spontaneous hemorrhage in patients with cirrhosis and renal failure, and angiosarcomas. 1 2 3 The presentation of hemorrhagic cholecystitis can be easily confused with a variety of more common diagnoses. As with any form of acalculous cholecystitis, hemorrhagic cholecystitis can present with leukocytosis and unexplained fever. Clots can form inside the gallbladder, leading to gallbladder distention and right upper quadrant pain, possibly leading to perforation into the abdomen. If this occurs, the patient could present with symptoms of frank peritonitis. Blood could flow freely and enter the bowel lumen, presenting with hematemesis or melena. Finally, it can present with signs of obstructive jaundice. 4

Initially, the patient in this case only presented with fever and leukocytosis. Given his history and presentation, hemorrhagic cholecystitis was not suspected immediately. The patient in this case did have hemorrhage into the gallbladder and out into the immediate peritoneal cavity. However, he did not display any signs of peritonitis likely due to the containment of the bleeding to just the right upper quadrant and the gallbladder itself. The cystic duct was completely obstructed by clot formation, preventing hemorrhage into the intestinal lumen. Consequently, our patient did not experience any hematemesis or melena which would have signaled the presence of a hemorrhage. His exam was also normal until hospital day #2, when his INR jumped significantly. We believe that the starting of anticoagulation triggered his hemorrhage, which was then exacerbated when his INR increased. At the time of discharge, there was no clear explanation for why his INR had suddenly increased. Although we initially suspected a previously unknown liver disease as a cause for his sudden increase in INR, none of his laboratory work and imaging showed signs of significant cirrhosis. He was referred to follow-up with outpatient hepatology for further evaluation.

Hemorrhagic cholecystitis is associated with high morbidity and mortality rates, particularly when it is complicated by perforation, necrosis, and potentially massive hemorrhage. Empiric antibiotic therapy should be initiated as secondary infection by enteric pathogens is common in the setting of acute acalculous cholecystitis. 5 Definitive treatment for cholecystitis is typically a cholecystectomy, although a percutaneous cholecystostomy may be performed for acute management in a patient with significant comorbidities preventing a cholecystectomy. If necessary, a delayed cholecystectomy can then be performed. 6 7 Percutaneous cholecystostomy is not indicated in the presence of gallbladder gangrene or perforation, but this may be challenging to determine through imaging alone. 8

In summary, hemorrhagic cholecystitis is a rare finding but its complications are often fatal. Because its presentation may be similar to many other conditions, a high index of suspicion is necessary for its diagnosis, particularly in the setting of an acutely ill patient with other comorbidities or on anticoagulation. Timely diagnosis is important to allow for early surgical management.

## Conclusion

The above case presents a rare presentation of hemorrhagic cholecystitis secondary to warfarin use for deep vein thrombosis treatment. Although rare, this disease entity should be considered in patients presenting with abdominal pain who are on anticoagulation. Early diagnosis and treatment can improve outcomes.
